# Comparative genome analysis of lignin biosynthesis gene families across the plant kingdom

**DOI:** 10.1186/1471-2105-10-S11-S3

**Published:** 2009-10-08

**Authors:** Zhanyou Xu, Dandan Zhang, Jun Hu, Xin Zhou, Xia Ye, Kristen L Reichel, Nathan R Stewart, Ryan D Syrenne, Xiaohan Yang, Peng Gao, Weibing Shi, Crissa Doeppke, Robert W Sykes, Jason N Burris, Joseph J Bozell, (Max) Zong-Ming Cheng, Douglas G Hayes, Nicole Labbe, Mark Davis, C Neal Stewart, Joshua S Yuan

**Affiliations:** 1 Department of Plant Pathology and Microbiology, Texas A&M University, College Station, TX, USA; 2 Institute for Plant Genomics and Biotechnology, Texas A&M University, College Station, TX, USA; 3 Department of Plant Sciences, University of Tennessee, Knoxville, TN, USA; 4 National Renewable Energy Laboratory, Golden, CO, USA; 5 Oak Ridge National Laboratory, Oak Ridge, TN, USA; 6 Department of Biosystems Engineering and Soil Sciences, University of Tennessee, Knoxville, TN, USA

## Abstract

**Background:**

As a major component of plant cell wall, lignin plays important roles in mechanical support, water transport, and stress responses. As the main cause for the recalcitrance of plant cell wall, lignin modification has been a major task for bioenergy feedstock improvement. The study of the evolution and function of lignin biosynthesis genes thus has two-fold implications. First, the lignin biosynthesis pathway provides an excellent model to study the coordinative evolution of a biochemical pathway in plants. Second, understanding the function and evolution of lignin biosynthesis genes will guide us to develop better strategies for bioenergy feedstock improvement.

**Results:**

We analyzed lignin biosynthesis genes from fourteen plant species and one symbiotic fungal species. Comprehensive comparative genome analysis was carried out to study the distribution, relatedness, and family expansion of the lignin biosynthesis genes across the plant kingdom. In addition, we also analyzed the comparative synteny map between rice and sorghum to study the evolution of lignin biosynthesis genes within the *Poaceae *family and the chromosome evolution between the two species. Comprehensive lignin biosynthesis gene expression analysis was performed in rice, poplar and *Arabidopsis*. The representative data from rice indicates that different fates of gene duplications exist for lignin biosynthesis genes. In addition, we also carried out the biomass composition analysis of nine *Arabidopsis *mutants with both MBMS analysis and traditional wet chemistry methods. The results were analyzed together with the genomics analysis.

**Conclusion:**

The research revealed that, among the species analyzed, the complete lignin biosynthesis pathway first appeared in moss; the pathway is absent in green algae. The expansion of lignin biosynthesis gene families correlates with substrate diversity. In addition, we found that the expansion of the gene families mostly occurred after the divergence of monocots and dicots, with the exception of the C4H gene family. Gene expression analysis revealed different fates of gene duplications, largely confirming plants are tolerant to gene dosage effects. The rapid expansion of lignin biosynthesis genes indicated that the translation of transgenic lignin modification strategies from model species to bioenergy feedstock might only be successful between the closely relevant species within the same family.

## Introduction

Lignin is one of the most important biomolecules in vascular plants and is uniquely involved in the structure support, water transport, and other functions [1,2]. The emergence of lignin during evolution is believed to be a crucial adaptation for plants to live on land. Plant cell walls are composed of cellulose, hemicellulose, lignin and cell wall proteins [3-6]. As a major component of plant cell wall, lignin cross-links the cellulose and hemicellulose to form a mesh-like structure giving mechanical strength necessary for upright stature [5]. Moreover, lignin is highly hydrophobic, which allows it to play an essential role in water transport and to serve as a major component of vascular tissue [7,8]. In addition, lignin is involved in a variety of biological functions including plant defense and abiotic stress resistance [9,10].

The distribution of lignin among plant tissues and across plant species is highly relevant to its function. Lignin is mainly deposited in the secondary cell wall; the primary cell wall generally does not contain lignin [4,5]. Woody plant vascular tissue is highly ligninized, presumably because of long-distance water transport and required mechanical strength [7]. Historically, lignin was thought to be a unique component of vascular plants; however, lignin or lignin-like molecules have recently been found in bryophytes and red algae [11,12]. Nevertheless, it is generally believed that green algae do not contain lignin. Even though there is considerable variation in content and composition of lignin in the plant kingdom, very few studies have systemically analyzed the evolution and function of lignin biosynthesis gene families across the kingdom. The recent availability of genome sequences for several plant species enabled the comparative genomic analyses to make inferences about the evolution of lignin biosynthesis gene families across the plant kingdom [13-16].

There are two major steps of lignin biosynthesis in plants: monolignol biosynthesis and the subsequent cross-linking of lignin monomers to form polymers, which then connects them to hemicellulose and cellulose. Most of the current research has focused on monolignol biosynthesis. Peroxidases and laccases are believed to be involved in dimerization and cross-linking of the monoligols, but more detailed mechanisms have yet to be unveiled [17]. The biochemical pathways of monolignol biosynthesis are highly conserved throughout vascular plants, and most of the enzymes in the monolignol biosynthesis pathway have been identified and characterized. Lignin biosynthesis starts with the amino acid phenylalanine, where phenylalanine ammonia lyase (PAL) catalyzes the deamination reaction to produce cinnamic acid. Cinnamic acid is then converted into p-coumaric acid by cinnamate 4-hydroxylase (C4H). The further hydrolation and methylation steps are branched and complicated. Recent work has revealed a predominant monolignol biosynthesis pathway, however, other derivative compounds are also produced, and additional pathway components cannot be ruled out [18]. The complexity of the lignin biosynthesis pathways is attributed to several multifunctional enzymes, and these enzymes also correspond to diverse gene families. For example, COMT and F5H are both multifunctional enzymes. F5H controls the balance of guaiacyl (G) monolignol to syringyl (S) monolignol and can act on multiple substrates, including ferulic acid, coniferaldehyde and coniferyl alcohol. COMT is the enzyme catalyzing O-methylation of an even more diverse group of substrates, including caffeic acid, 5-hydroxy-coniferaldehyde, caffeyl alcohol, 5-hydroxyl-feruloyl CoA, and 5-hydroxy-coniferyl alcohol [18]. Despite the progresses in biochemical studies, it is not clear how the complicated biochemical pathway evolved and if the substrate diversity actually is relevant to the gene family expansion.

Lignin biosynthesis has been subject to intensive study during the past two decades, mainly driven by the significant needs in forage and biofuel industries [3]. Lignin content and composition are closely relevant to the digestibility of plant cell walls. The elucidation of the lignin biosynthesis pathway has enabled various strategies for modifying lignin content and composition to improve the digestibility of forage and for biofuel production [3]. The reduction of lignin content can greatly increase the saccharification efficiency of biomass processing for lignocellulosic biofuels [19]. In fact, a top priority of lignocellulosics research is to develop greater levels of control over feedstock cell wall structure leading to improved biopolymer process streams more suitable for conversion to chemicals and fuels [20]. Decreased lignin content has been shown to render two major advantages for potential bioenergy feedstocks. First, plant biomass with decreased lignin is more readily saccharified. Reducing lignin production can derive more accessible cellulose for downstream chemical and enzymatic treatment in woody species and several other dicot species including *Arabidopsis*, *Medicago*, tobacco, and poplar [3,19,21-27]. Second, reduced lignin biosynthesis can lead to more carbon allocated to sugar synthesis, and thus, might result in higher amounts of cellulose and hemicellulose production [21,23]. In addition to lignin content, modification of lignin composition is also believed to be able to alter the digestibility and saccharification efficiency for lignocellulosics [28]. Despite the progresses in biochemical analysis and genetic modification, many questions regarding the evolution and function of lignin biosynthesis gene families across species still remain unanswered. Very few studies have been carried out to analyze the relatedness among lignin biosynthesis genes, which significantly impacts how far we can translate the results from one species to another.

The study of evolution and function of lignin biosynthesis genes has two implications. First, our analysis provides crucial insights into the fundamental understanding of the evolution of land plants because of lignin's essential role in adaptation to land growth. The lignin biosynthesis pathway provides a perfect model for studying the evolution of a biochemical pathway at the systems level [5,29]. Systems biology should be a powerful tool to enable better understanding the evolution of biological networks [30]. The study of lignin biosynthesis evolution will help researchers determine if coordinative evolution of biochemical pathways exists, and how the different enzymes for producing a group of important molecules such as lignin might have evolved together. Another goal is to help to elucidate the evolution of land-adaptation for the vascular plants. We will address questions about the first appearance of lignin biosynthesis and patterns of evolution among diverse taxa. We will also examine the main driving force for the evolution of the lignin biosynthesis pathway. Second, this research should help guide genetic modification strategies from model species to bioenergy feedstocks. It is important to know the evolutionary relatedness and to better understand the functional conservation of lignin biosynthesis genes among taxa to optimally design next-generation bioenergy feedstocks with modified lignin.

In order to address the aforementioned questions, we carried out a comprehensive comparative genome analysis of lignin biosynthesis gene families. The comparative genome analysis is complimented by a gene expression study and the biomass composition analysis for *Arabidopsis *mutants. We first collected all of the lignin biosynthesis genes from 14 plant species and one symbiotic fungal species. The species selection covered both monocot and dicot as well as both higher plants and lower plants. The analysis revealed that the complete lignin biosynthesis pathway first appeared in moss. With moss (and probably red algae) as the first turning point for the existence of a complete lignin biosynthesis pathway, the birth of the F5H gene family in angiosperms might represent a second turning point of the lignin biosynthesis evolution. The phylogenic analysis also revealed that the expansion for lignin biosynthesis gene families mainly happened after the speciation between monocot and dicot. A certain level of coordinative evolution of the lignin biosynthesis pathway was found. The synteny map revealed high conservedness of rice and sorghum lignin biosynthesis genes, which could help to guide the lignin modification across the monocot species for bioenergy feedstock improvement. Gene expression profiling was carried out to analyze the regulation of lignin biosynthesis genes in response to various stresses, which helped to infer different fates for gene duplications. The biomass composition analysis of *Arabidopsis *lignin biosynthesis mutants revealed that phylogenetic analysis across distant species could not be used to guide the lignin modification for feedstock improvemnt due to the recent and rapid evolution of the gene family. The research highlighted the need of choosing suitable model species to develop the gene-specific lignin modification strategies.

## Results

### Protein sequence collection and classification for lignin biosynthesis gene families

**Figure 1:**
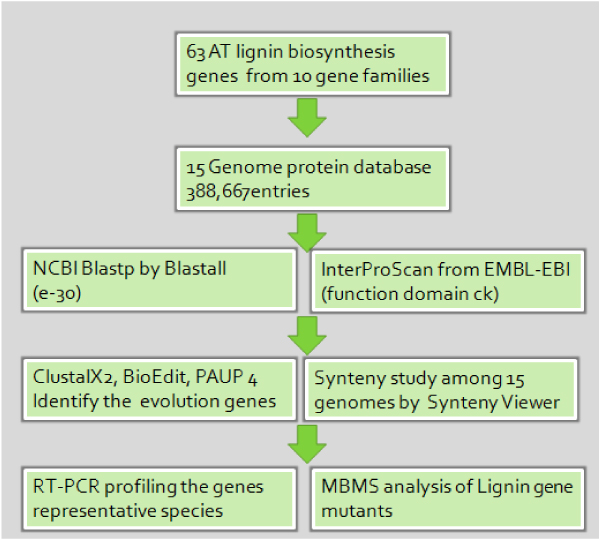
**Schema of the experiment**.

The first step of our analysis was to identify all lignin biosynthesis genes from fifteen species with a two-step schema as shown in Figure 1. The monolignol biosynthesis pathway has been well characterized with ten enzymes involved in the conversion of phenylalanine into different monolignol molecules [1,18]. Each enzyme is encoded by a gene family in most of the plant species [1,18]. Protein sequences for the 10 enzymes were first retrieved from *Arabidopsis *database based on the previous biochemistry and genome analysis [31-34]. A total of 63 protein sequences were retrieved and the functional domains were identified for each gene family. The protein sequences from *Arabidopsis *served as the base for the lignin biosynthesis gene identification.

The first step for the gene identification was to find the candidate lignin biosynthesis genes with similarity search. Blastp analysis was carried out to search against a database with a total of 388,667 annotated protein sequences from fifteen species. For most of the gene families, an E value cutoff of e-30 was used. The E value for the CCR gene family cutoff was e-24; and the E value cutoff for COMT was e-08. With the aforementioned E value cutoff, a total of 6564 homologous sequences were identified as shown in Table 1. The second step of gene identification was based on the function domain. IntroProScan was used to analyze the 6564 candidate genes and the protein sequences not containing the well-defined functional domain for lignin biosynthesis were removed. After the second step, a total of 818 genes were retrieved and these are summarized in Table 1 and Table 2.

**Table 1:** Identification of lignin biosynthesis genes

Enzymes	Domain investigated	E-value	NSH****	NGH****	NGH/NSH (%)****
C3H	PTHR19383:SF44	1.00E-30	993	14	1.5
F5H	PTHR19383:SF46	1.00E-30	990	15	1.5
C4H	PTHR19383:SF33	1.00E-30	530	18	3.4
4CL	PTHR11968:SF43	1.00E-30	2212	120	5.4
CAD	PTHR11695:SF38	1.00E-30	1022	110	10.8
HCT	PF02458	1.00E-30	279	37	13.3
CCOAMT	PTHR10509	1.00E-30	61	55	90.2
CCR*	PTHR10366:SF9	1.00E-24	207	207	100.0
PAL**	PF00221/TIGR01226	1.00E-30	55	55	100.0
COMT***	PIRSF005739	1.00E-08	187	187	100.0
Total	11	Na	6536	818	Na

Summary results from different steps of gene identification including Blastp and InterproScan analysis are presented along with the functional domains used for protein identification.

* We used E value cutoff of e-24 for CCR gene identification, which is supported by the functional domain data.

**The identification of PAL genes involved two domains.

*** The E value cutoff for COMT gene identification is e-08, which is supported by the functional domain data.

****Number of sequence homologs (NSH), Number of gene homologs (NGH), and Pentage of the NGH in the NSH were included.

CAD: Cinnamyl alcohol dehydrogenase

PAL: L-Phenylalanine ammonia-lyase

CCOAMT: Cinnamoyl CoA O-methyltransferase

COMT: Caffeic acid *O*-methyltransferase

4CL: 4-Coumarate:coenzyme A(CoA) ligase

CCR: Cinnamoyl-CoA reductase

C4H: Cinnamate-4-hydroxylase

HCT: Hydroxycinnamoyl transferase

C3H: 4-Coumarate 3-hydroxylase

F5H: Ferulate 5-hydroxylase

**Table 2:** Summary of the species and genome resources used in the study

Sample	Common Name	Name	Classification	Total transcripts	URL reference for genome
1	mushroom	*Laccaria bicolor*	Symbiotic fungus	19,036	http://genome.jgi-psf.org
2	microalgae	*Ostreococcus tauri*	Prasinophyte	*7725*	http://genome.jgi-psf.org
3	microalgae	*Ostreococcus lucimarinus*	Prasinophyte	*7,651*	http://genome.jgi-psf.org
4	microalgae	*Ostreococcus RCC809*	Prasinophyte	*7773*	http://genome.jgi-psf.org
5	diatom	*Phaeodactylum tricornutum*	Bacillariophyte	10025	http://genome.jgi-psf.org
6	diatom	*Thalassiosira pseudonana*	Bacillariophyte	11390	http://genome.jgi-psf.org
7	green algae	*Chlamydomonas reinhardtii*	Chlorophyte	14,598	http://genome.jgi-psf.org
8	alga	*Volvox carteri*	Chlorophyte	15,544	http://genome.jgi-psf.org
9	moss	*Physcomitrella patens*	Bryophyte	35938	http://genome.jgi-psf.org
10	spike moss	*Selaginella moellendorffii*	Lycophytes	34,697	http://genome.jgi-psf.org
11	Sorghum	*Sorghum bicolor*	Monocot	34496	http://genome.jgi-psf.org
12	Arabidopsis	*Arabidopsis thaliana*	Dicot	32016	http://www.arabidopsis.org/
13	Poplar	*Populus trichocarpa*	Dicot	45555	http://genome.jgi-psf.org
14	Rice	*Oryza sativa*	Monocot	67,393	http://rice.plantbiology.msu.edu/
15	Medicago	*Medicago truncatula*	Dicot	44830	http://www.medicago.org/
Total				388,667	

As shown in Table 1, C3H is the smallest gene family, and it is highly conserved. From the perspective of gene family size, gene function, and sequence similarity, C3H might be the most conserved gene family among the ten families analyzed. However, from the perspective of gene family evolution, C4H might be the most conserved gene family because the phylogenetic analysis revealed no clear separation between monocot and dicot C4H genes indicating that most of the gene family expansion actually happened before the divergence between monocot and dicot (Additional File 1). Four gene families, CCR, COMT, CAD and 4CL had greater than 100 members across the 15 species. COMT is the least conserved gene family, considering both gene family size and the low similarity among the genes from different species. COMT, CAD, CCR and 4CL are also the more expanded as compared to the other several gene families. Among these, CCR is the most expanded gene family with 207 members across species representing the largest lignin biosynthesis gene family in several species.

The distribution of the lignin biosynthesis genes across the fifteen species is summarized in Table 3. *Physcomitrella *(moss) is the first species with the complete lignin biosynthesis pathway with the exception of F5H. Several gene families first appeared in moss. F5H only exists in the spermatophyte. CAD and CCoAMT genes were found in several unicellular algae species, and 4CL and CCR were also found in the a few algae species. PAL first appeared in *Volvox*, one of earliest multicellular plant species. Several other lignin biosynthesis genes were also found in *Volvox*. Recent studies indicated that red algae also contains lignin; however, we were not able to include a red algal species because no whole genome sequence is available at the time of this study [12,13].

**Table 3:** Summary of number of lignin biosynthesis genes in each gene family across the 15 species studied.

Species	CAD	CCoAMT	4CL	CCR	PAL	C4H	HCT	COMT	C3H	F5H	Total
*O. tauri*	3	1	0	0	0	0	0	0	0	0	4
*O. RCC809*	2	1	0	0	0	0	0	0	0	0	3
*O. lucimarinus*	3	1	0	0	0	0	0	0	0	0	4
*Phaeodactylum*	1	1	1	2	0	0	0	0	0	0	5
*Thalassiosira*	0	0	1	0	0	0	0	0	0	0	1
*Chlamydomonas*	4	2	0	4	0	0	0	0	0	0	10
*Laccaria bicolor*	2	1	5	0	2	0	0	0	0	0	10
Volvox	3	2	0	1	1	0	0	0	0	0	7
Physcomitrella	4	2	11	7	14	4	4	3	1	0	50
Spike moss	18	8	26	29	2	2	6	28	2	0	121
Arabidopsis	9	7	13	7	4	1	1	16	3	2	63
Medicago	21	4	10	18	4	1	6	26	1	3	93
Sorghum	14	7	15	44	8	3	4	41	2	3	141
Rice	5	11	16	55	14	4	9	38	1	3	157
Poplar	21	7	22	40	6	3	7	35	4	4	149
Total	110	55	120	207	55	18	37	187	14	15	818

### The relatedness of lignin biosynthesis genes across the species

**Figure 2:**
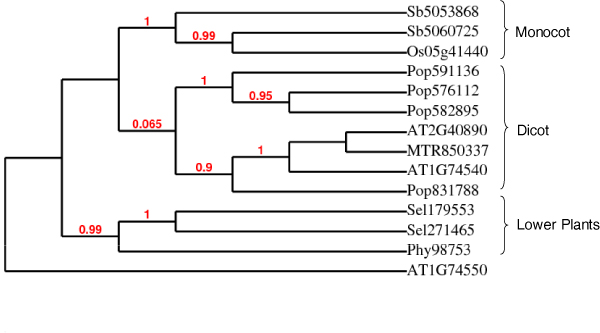
**Phylogenetic analysis of C3H genes from seven species**. The figure shows a clear distinction of three subgroups with the exception of one *Arabidopsis *genes. The groups include lower plant species (moss and spike moss), monocots (sorghum and rice), and dicots (poplar, *Medicago*, and *Arabidopsis*). The species is indicated by the first two or three letter of the gene name as follows; AT: *Arabidopsis*; MTR: *Medicago*; SEL: spike moss; OS: rice; POP: poplar; PHY: *Physcomitrella*; SB: sorghum.

**Figure 3:**
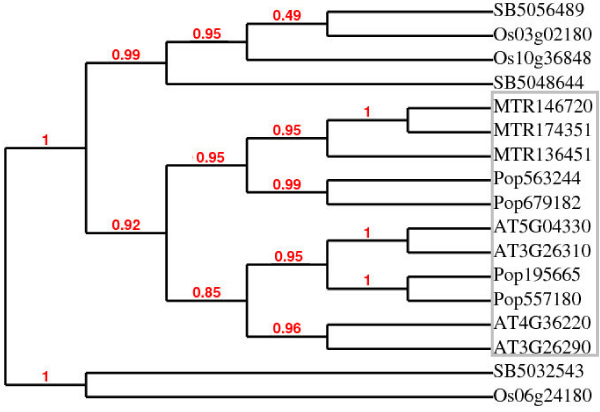
**Phylogenetic analysis of F5H genes from five species**. The analysis revealed clear separation between monocot and dicot species. The species is indicated by the first two or three letter of the gene names as follows: AT: *Arabidopsis*; MTR: *Medicago*; OS: rice; POP: poplar; SB: sorghum. The shaded area is a dicot group of genes. The gray box includes all dicot genes.

Phylogenetic analysis showed that lignin biosynthesis genes from seed plants share clades and the lignin biosynthesis genes from lower plants share clades. A distinction can also be found between monocot and dicot species with the exception of the C4H gene family. Phylogenetic analysis as shown in Additional File 2 and 3 suggested that genes from spermatophytes and lower plants were clustered into separate groups. The same phenomenon was also found between monocots and dicots. C3H is considered as one of the most conserved gene families and appeared first moss. As shown in Figure 2, the phylogenetic analysis of C3H genes across different plant species clearly reveals three groups: lower plants (plants with no seeds), monocots, and dicots. The evolution of F5H genes is still controversial, because of the recent finding of S lignin in spike moss and *Ginkgo biloba*. However, it is expected that the F5H genes in angiosperms and other F5H genes evolved through convergent evolution. As shown in Figure 3, our phylogenetic analysis reveals that the F5H genes are distinct between monocots and dicots.

### The expansion of lignin biosynthesis gene families during evolution

The relatedness of the lignin biosynthesis genes is directly connected to gene family expansion in evolution. As shown in Figure 3, most lignin biosynthesis gene families experienced rapid and recent duplications. No more than five gene family members exist in any single species earlier than *Physcomitrella *(moss). In contrast, moss has 26, 29, and 28 copies of 4CL, CCR, and COMT, respectively, which makes moss a dividing line and turning point for the evolution of lignin biosynthesis. These results corroborate the findings of the *Physcomitrella *genome project [13].

Another turning point for the lignin biosynthesis evolution is the birth of F5H genes. F5H converts G monolignol to S monolignol. As aforementioned, the F5H gene homologs in our study were not found in lower plants, but existed in all five angiosperm species as shown in Table 1. The evolution of F5H genes is complicated. It is believed that F5H genes evolved after the divergence between angiosperm and gymnosperm. No S lignin has been found in any gymnosperm species. However, there is a recent report that S lignin does exist in lycophytes, such as spike moss and Ginkgo. The F5H gene in spike moss evolved through convergent evolution and does not share a high similarity with the rest of the F5H genes in our analysis. The fact that we did not identify the spike moss F5H gene through homology-based methods confirms the convergent evolution of S lignin biosynthesis. Considering that the gene family is relatively new, F5H has very few copies in each of the 5 investigated gymnosperm genomes.

### Horizontal gene transfer between symbiosis Laccaria and plant species

**Figure 4:**
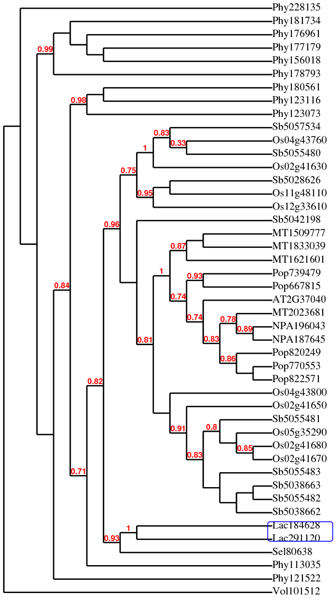
**Phylogenetic analysis of PAL genes from eight species including *Laccaria bicolor***. The species is indicated by the first two letters of the gene names as described in Figure 2. AT: *Arabidopsis*; MTR: *Medicago*; SEL: spike moss; OS: rice; POP: poplar; PHY: *Physcomitrella*; SB: sorghum; LAC: mushroom; VOL: *Volvox*. The two *Laccaria *genes were circled by a blue box.

Horizontal gene transfer (HGT) refers to the process by which an organism incorporates genetic material from another without being the offspring of that organism. We found four cases of potential HGT among plant species and the fungal species. CAD, CCoAMT, 4CL, and PAL were all found in *Laccaria bicolor*, a symbiotic fungus associated with pines and spruces. As shown in Figure 4, two PAL gene members in *Laccaria *(LA184628 and LA291120) share clades with a spike moss PAL gene indicating that HGT might be an early driver in its evolution. It is important to consider the origin of lignin during the evolution when discussing the direction of HGT.

### Chromosomal location of genes and synteny between rice and sorghum

Comparative synteny mapping across species allows the observation of how chromosome evolution occurred and how the orthologs from different species evolved in relation to one another. We particularly focused on the comparative synteny analysis of rice and sorghum for two reasons. First, most bioenergy crops are monocot species. The study of comparative synteny mapping between two monocot model species allows for the understanding about how genes evolve within monocot species and thus allows better identification of genes to target for genetic modification. In fact, rice and sorghum also represent two types of carbon metabolism: C3 and C4. Most bioenergy feedstocks are C4 warm-season grasses. Second, the lignin biosynthesis genes experienced rapid gene evolution as aforementioned. Both rice and sorghum belongs to the *Poaceae *family, which allowed us to identify more orthologs.

**Figure 5:**
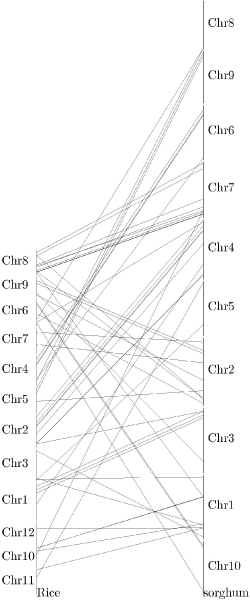
**Collinearity map for rice and sorghum based on the orthologs of lignin biosynthesis genes**.

The orthologs from ten lignin biosynthesis gene families in both rice and sorghum were mapped and their corresponding chromosome locations are summarized in Table 4 and Figure 5. For C3H, there is only one copy in sorghum (chromosome 9) and rice (chromosome 5), respectively. In contrast, CCR has more orthologs, with 12 loci found on 7 sorghum and 9 rice chromosomes, respectively. Collinearity mapping is a more reliable phylogenetic tool than only similarity based synteny. At the whole-genome level, there is some collinearity between rice and sorghum. Among the 60 investigated loci from 10 lignin biosynthesis gene families, 49 have collinearity (82%). The comparative synteny map also revealed the expansion and inversion of some chromosome regions in sorghum.

**Table 4:** Distribution of the ten lignin biosynthesis gene families in rice and sorghum chromosomes

Gene Family	Number of Chromosome in Sorghum	Number of Genes in Sorghum	Chromosome Name in Sorghum	Number of Chromosome In Rice	Number of Genes in Rice	Chromosome Name in Rice
C3H	1	2	9	1	2	5
C4H	3	4	2,3,4	3	4	1, 2, 5
CAD	3	5	4,6,7	4	5	2,4,8,10
CCOAMT	3	3	2,7,10	3	3	6,8,9
F5H	3	3	1,2,5	3	3	3,6,10
HCT	3	4	2,4,6	4	4	2,6,4,9
PAL	3	7	1,4,6,	4	7	2,4,11,12
4CL	6	12	1,2,3,4,7,10	7	12	1,2,3,6,7,8,10
COMT	6	9	2,3,4,5,7,8	6	9	1,2,4,6,8,11
CCR	7	12	1,2,3,4,7,9,10	9	12	1,2,3,5,6,7,8,9,10
Total	38			44		

The data summarize how many genes exist for each family and how many chromosomes contain these genes.

### Stress-induced gene expression of lignin biosynthesis genes

**Figure 6:**
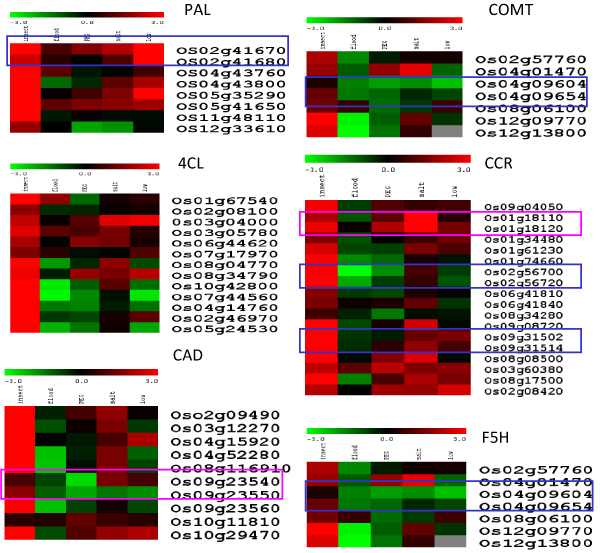
**Cluster analysis of lignin biosynthesis genes under different treatments**. The logarithm 2 transformed ratios were used, and the color schema is as shown in the figure. The gene ID is shown on the right and the different treatments including insect, flood, salt, PEG (for osmotic stress), and low temperature are labeled. The blue box indicates that the paralog genes share a similar gene expression pattern (possible RR mode), while the pink box indicates that the paralog genes have evolved different gene expression patterns (possible RN mode).

Real-time PCR of six large lignin biosynthesis gene families in rice, poplar and *Arabidopsis *was performed to observe the fate of gene duplication. Figure 6 shows the gene expression pattern for rice lignin biosynthesis genes. We use rice as the model species in this article to analyze the potential function and gene fates for lignin biosynthesis genes. Insect treatment induced the most significant changes in gene expression. Other abiotic stress treatment induced different expression patterns among different genes as shown in the figure.

The most important observation was the modes of evolution for the recently duplicated genes. Changes in gene or protein motifs can have multiple modes. Gene copies can have retention (R) of the original motif organization and function. Degeneration (D) mode suggests loss of one or more motifs and functions. Neofunctionalization (N) mode describes a new function or motif in a gene copy. Thus, the schema of RN, RD, NN, DD has been proposed to describe the major fates for the duplicated genes. Duplicated genes therefore would be expected to develop a new function (RN) or degenerate during evolution because of gene dosage effects (RD) [35-38]. However, it appears that RR is the mode for several pairs of lignin biosynthesis genes based on the gene expression study as shown in Figure 6. Since most of the lignin biosynthesis genes maintain the core domain for catalysis, we maintain that no motif or organization changes have occurred in the enzymes. Gene expression levels might reflect their functionality to a certain degree. Similar gene expression patterns might indicate no gene function divergence (RR) and a distinct gene expression pattern might indicate that new function has evolved (RN). As shown in Figure 6, for the several paralogs we analyzed, there are potentially more genes in RR mode than RN mode. Similar phenomena were found in all three species according to the gene expression analysis. These results shed light on the fate of gene duplications in plants, particularly for genes in the very important lignin biosynthesis pathway.

### Biomass composition analysis of Arabidopsis mutants

**Figure 7:**
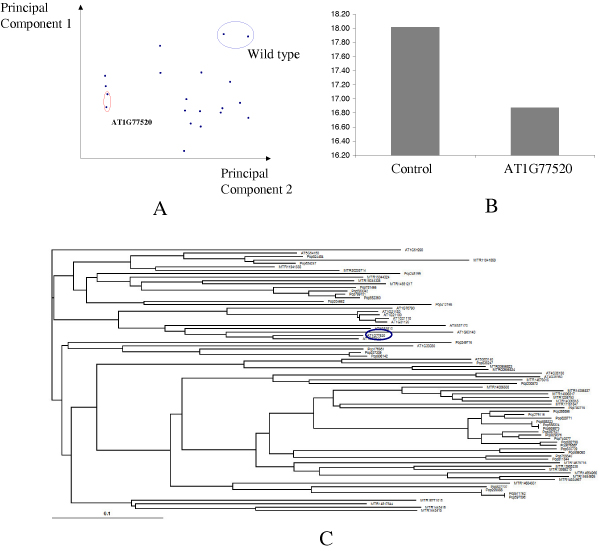
**Biomass composition analysis and phylogenetic analysis of the COMT gene family**. Panel A shows principle component analysis (PCA) of the MBMS data. Panel B shows the wet chemistry analysis of the sample. Panel C shows phylogenetic analysis of COMT gene families in all dicot species.

In order to examine how functional and comparative genomics information can be used to guide the genetic modification, we carried out the biomass composition analysis for nine *Arabidopsis *mutants. The choice of the mutants largely depended on the availability of the homozygous mutants in the ABRC (*Arabidopsis *Biological Resource Center). The result for biomass analysis is as shown in Figure 7. The py-MBMS (pyrolysis-molecular beam mass spectrometer) was first used to screen mutants based on the analysis of mass spectra of biomass after pyrolysis. Principal components analysis (PCA) showed that one mutant was particularly different from the wild-type and the rest of the samples (Figure 7A). The wet-chemistry experiments were then carried out to analyze different mutants for their carbohydrate and lignin contents and composition. The At1G77520 mutant had the lowest lignin content, with about 20% lignin reduction (Figure 7B). Both MBMS and wet chemistry data confirmed that AT1G77520 is the mutant with largest lignin reduction among the ten mutants. AG1G77520 was not one of the conserved genes in dicot species (Figure 7C).

## Discussion

This study represents one of the first comprehensive analyses of the evolution of lignin biosynthesis across the plant kingdom, because we have taken advantages of the recently available plant genome sequences. We have found several important features of lignin biosynthesis evolution and subsequently apply these features to develop strategies for lignin modification.

### Lignin biosynthesis gradually evolved

It is clear from the results that lignin biosynthesis evolved gradually. Some lignin biosynthesis genes appeared early in plant evolution, and these gene families included CAD, CCoAMT, CCR, and 4CL. PAL first appeared in *Volvox*, a multi-cellular microscopic chlorophyte that contains one cell type. Several gene families including C3H, C4H, COMT and HCT all appeared first in moss among the 14 plant species studied. The existence of lignin biosynthesis genes correlates with the distribution of lignin across the plant kingdom. It is generally believed that green algae do not contain lignin and indeed we found no complete lignin biosynthesis pathway in any of the algae or diatom species. However, the recent finding of lignin in red algae has expanded the distribution of lignin across the plant kingdom [12]. Even though the existence of lignin in moss is still controversial, it is generally believed that moss contains 'uncondensed' monolignol-like molecules [11].

The discovery of lignin in red algae also poses an important question regarding the origin of the lignin biosynthesis pathway. The current lignin biosynthesis pathways could come from two origins. On one hand, it is possible that the lignin biosynthesis pathway in embryophytes existed before the divergence of green and red algae more than 1 billion years ago [39,40]. Green algae might have lost the capacity to produce lignin during evolution. This is also supported by the fact that some lignin biosynthesis genes, but not the complete pathway were found in green algae species. If so, HGT between symbiotic fungi and plants might be in the direction of plants to fungi. However, it could also be possible that red algae and land plants evolved lignin biosynthesis pathway through convergent evolution. The lignin biosynthesis pathway in land plants might evolve from HGT of some important lignin biosynthesis genes from microbes, but these scenarios are highly speculative. It is therefore important to analyze lignin biosynthesis genes from red algae species when their genomes are available.

Despite these various speculations about the origin of lignin biosynthesis, there are clearly two turning points for its evolution. Three pieces of evidence supports moss as the first turning point – the host for "original lignin". First, across the 14 plant species, moss is the first species with nine lignin biosynthesis gene families, all of which represent the entire lignin biosynthesis pathway. Second, as compared to the green algae species, there is a sudden expansion of lignin biosynthesis gene family members in moss. Third, lignin and monolignol-like molecules were found in some red algae and moss species, but not in green algae. The results indicated that moss is a basal linage of land plants and a key evolution point for vascular development [41]. The second turning point for lignin biosynthesis is the birth of the F5H gene family. F5H genes convert G monolignol to S monlignol. The recent finding of S lignin in lycophyte and Ginkgo complicates the assumption that F5H only exists in angiosperm. However, our data support the assertion that lycophyte F5H does not share the same lineage as that of the angiosperms. F5H genes from angiosperms, lycophyte, and Ginkgo could all be the products of convergent evolution. F5H from angiosperms might represent the tipping point responsible for the evolution of cell wall structure diversity allowing for tall trees and various plant morphology characterized by angiosperms.

### The coordinative evolution of lignin biosynthesis

In addition to the gradual evolution of the lignin biosynthesis, a certain level of coordination can be found in its evolution. As previously discussed, among the ten lignin biosynthesis gene families, four of these did not appear earlier than moss. All of the gene families in lignin biosynthesis did not experience a significant gene family expansion except in embryophytes. In this study, moss can actually be considered the plant when and where coordinative evolution of lignin biosynthesis begins. The coordinative evolution of lignin biosynthesis pathway correlates with the evolutionary systems biology theory that biological pathways can be evolved at the systems level rather than individually [5,29,30].

### Substrate diversity and gene family expansion

Despite the coordinative evolution of lignin biosynthesis genes, different gene families experienced various levels of expansion. We found a high correlation between substrate diversity and gene family expansion. A detailed examination of lignin biosynthesis pathways led to the classification of lignin biosynthesis genes into three groups based on the substrate availability [18]. The first group includes 4CL, CCR, COMT and CAD, all of which can convert multiple substrates into different products. The second group includes PAL, C4H, C3H, HCT, and CCoAMT, which specifically convert one or two substrates into products. The third group is the recently evolved F5H gene, which coverts two or three substrates into products. A clear correlation between substrate diversity and the expansion of gene families can be found. Basically, 4CL, CCR, COMT, and CAD are the only four gene families with more than 100 members across the 14 plant species analyzed. F5H is relatively conserved with fewer gene family members – probably because it is a recently evolved gene family. The remaining gene families have between 14 and 60 members. Apparently, substrate diversity drives gene family expansion.

It should be noted that we selected the gene family members mainly on the basis of the conserved domain of function. Considering the substrate diversity and our limited understanding of lignin biosynthesis enzyme function, we may have overlooked some genes, which are actually involved in lignin biosynthesis. Not including these genes in the analysis could lead to biased conclusions.

### Fate of duplicated genes

In addition to the gene family expansion, the study also sheds light into the fate of the duplicated genes. The existence of a high percentage of RR mode indicates that plants could tolerate high gene dosage for lignin biosynthesis genes. Previous research in fungi indicated that growth-related genes are more sensitive to high gene dosage, and stress response genes are more tolerant to higher dosage [42]. A diverse fate of duplicated genes has been found in the previous analysis of Dof gene family, which indicated that plants might be more tolerant to the gene dosage effects compared with animals [43]. Our study echoes the results from these previous findings.

### Driving force for lignin biosynthesis evolution

Lignin biosynthesis gene families obviously experienced recent and rapid expansion. The expansion can be confirmed by the relatedness of the lignin biosynthesis genes, where phylogenetic analyses of most gene families contain three major clades: lower plants, monocots, and dicots. The phenomena indicates that the lignin biosynthesis gene family expansion in spermatophytes probably happened after the divergence between monocots and dicots 120 million years ago [44]. Many orthologs have been found between the monocots rice and sorghum, which indicates that some gene family expansion happened before their divergence. We also found many recent tandem duplications for rice and sorghum lignin biosynthesis genes; these are the genes without clear orthologs between species, indicating that lignin biosynthesis may still be under rapid evolution. However, not all lignin biosynthesis gene families are evolving rapidly. Some gene families, such as C4H, are rather conserved during evolution, which can also be seen by the phylogenetic analysis (Additional File 1), where no clear distinction can be found between monocot and dicot species.

It is difficult to delineate the driving forces for the recent expansion of lignin biosynthesis genes. According to the synteny map and phylogenetic analysis, even though some early whole genome duplication might account for gene family expansion, recent gene duplication is predominant.

Several factors could be involved in driving lignin biosynthesis evolution, including environmental adaptation and development of new structures and greater plant stature. We cannot exclude any one of them. However, other cell wall-related gene families like mananase are rather conserved across species, which indicates that growth and development might not be the most important driving force [45]. The ubiquitous over-expression of lignin biosynthesis genes in response to the insect treatment indicates that plant defense against insects and pathogens could be a major driving force for the recent duplication of lignin biosynthesis gene families. In fact, rapid gene family expansion has been observed in many defense-related gene families such as cytochrome P450s and terpene synthases [10]. Recent studies in fungi also found that stress-related genes tend to expand and disappear more dynamically as compared to the development-related gene families [42]. We therefore propose that biotic defense as a driving force for the recent and rapid expansion of lignin biosynthesis gene families.

### From evolution to function and lignin modification

C3H is one of the most conserved gene families among all the different lignin gene families and catalyzes a crucial reaction that cannot be substituted by other genes. A previous study in *Medicago *proved that down-regulation of C3H genes can produce the maximized effects on lignin content reduction as compared to other gene families [19]. The importance of C3H in lignin regulation is plausible because it is more conserved and potentially more important than other gene families. However, the mutants of C3H showed a severe developmental phenotype. We need to identify other genes as candidates for lignin modification. In particular, a gene-specific strategy needs to be developed. More importantly, we also want to know how the knowledge and strategies developed in one species can be applied to another.

We found that knocking down a COMT gene, AT1G77520, can reduce lignin content up to 20%, which makes the gene a perfect candidate gene for genetic modification. However, we found that the gene is not conserved among the three dicot species that we surveyed. It is actually a recently duplicated gene. The results indicate that phylogenetic analysis might not deliver the strategy to translate the findings from model species into bioenergy feedstocks if the two species are not closely related to one another. However, the existence of many orthologs between rice and sorghum suggests that a model species within the *Poaceae *family could serve the purpose of translational research for developing lignin modification strategies.

## Materials and methods

### Gene sequence collection, function domain comparison, and gene family member identification

Protein sequences for each of the ten gene families of the monolignol biosynthesis pathway were collected and analyzed as shown in Table 1. As the first step, a total of 63 protein sequences of the 10 gene families were retrieved from the *Arabidopsis *database http://www.arabidopsis.org/ and their protein function domains were examined with "InterProScan" from EMBL-EBI http://www.ebi.ac.uk/interpro/. The specific domain for protein identification is as shown in Table 1. In order to study the evolution of lignin biosynthesis genes, 15 genome sequences were selected. The species includes three parisnophytes, two chlorophytes, one lycophyte, one bryophyte and five spermatophyes. In addition, we also included two diatoms. Although the classification of diatoms is controversial, we chose to consider diatoms as plants [46]. In addition, we also included a symbiotic fungus species. The species selection reflects the current stage of available plant genomes as well as the range of lignin in plants. No lignin has been documented in the algae species and diatoms we selected. The existence of lignin in moss is also very controversial and it is generally believed that moss has lignin-like structures in their cell walls [11,47-49]. Lycophytes like *Selaginella moellendorffii *represent an ancient vascular plant with an independent origin of S lignin [50]. The remaining species include five higher plants with available genome sequence. Gene models for 13 out of 15 genomes were downloaded from DOE-JGI http://genome.jgi-psf.org as showed in Table 2. The *Arabidopsis *gene models were downloaded from TAIR http://www.arabidopsis.org/, and the rice gene models were downloaded from rice genome database http://www.tigr.org/tdb/e2k1/osa1.

A local blast database of the protein sequences from 15 genomes was formatted using formatDB http://www.ncbi.nlm.nih.gov/staff/tao/URLAPI/formatdb_fastacmd.html, and the initial protein sequence comparison was conducted using the Blastp algorithm at conservative e-value at e-30. Since the lignin biosynthesis gene family is large, it includes superfamilies like p450. InterProScan for protein domain comparison was carried out using default parameters. In order to derive the accurate prediction, no superfamily domain was used in the InterProScan search.

### Phylogenic analysis of the lignin biosynthesis genes

The protein sequences from above steps were edited by BioEdit http://www.mbio.ncsu.edu/BioEdit/BioEdit.html and functional domains were aligned through "do complete alignment" function using Clustal X2 under multiple alignment mode http://www.molecularevolution.org/cdc/software/clustalx/[51]. After the selected sequences were aligned, phylogenetic analysises were implemented with PAUP V4 software available from Sinauer Associates Inc http://paup.csit.fsu.edu/order.html using parsimony as well as phelogeny.fr http://www.phylogeny.fr/ using maximum likelihood [52]. For parsimony analysis, the phylogenetic trees were reviewed using TreeView http://taxonomy.zoology.gla.ac.uk/rod/treeview.html. The results from the two methods were compared and the trees produced with phelogeny.fr are presented, since the maximum likelihood method apparently produces more robust phylogenetic trees.

### Lignin biosynthesis gene distribution and synteny analysis

A synteny study of lignin biosynthesis gene families between rice and sorghum genome was conducted. The gene locations in rice were identified via the Rice Genome Annotation Project website http://rice.plantbiology.msu.edu/LocusNameSearch.shtml and locations for sorghum genes were identified by blast search at the DOE-JGI sorghum website http://genome.jgi-psf.org/Sorbi1/Sorbi1.home.html. A synteny map was drawn by software SyntenyView (Zhou, unpublished). The flowchart for the lignin biosynthesis gene family analysis is summarized in Figure 1.

### Plant growth, treatments, RNA extraction and real-time PCR experiments

Rice, *Arabidopsis *and poplar plant growth and abiotic stress treatments were performed as previously described [53]. Identical biological samples were used for our real-time PCR analysis of lignin biosynthesis genes [53]. Methods for real-time PCR primer design and quantification were as previously described [54-56]. Real-time PCR primers were designed by PrimerExpress and amplification efficiency was examined before quantification to ensure data quality [54-56]. The logarithm 2 transformed gene expression ratios were clustered using the MEV4.0 package.

### Biomass analysis with pyrolysis-molecular beam mass spectrometry (MBMS) analysis and wet chemistry

Both py-MBMS analysis and wet chemistry were carried out as previously described using the standard NREL protocol [57]. The *Arabidopsis *plants for mutant analysis were grown under the previously described conditions [10].

## Competing interests

The authors declare that they have no competing interests.

## Authors' contributions

JSY developed the project, supervised most of the work, interpreted the data, and drafted part of the manuscript. ZX performed most of the phylogenetic analysis and also drafted part of the manuscript. DZ carried out most of the real-time PCR analysis. JH assisted the phylogenetic analysis and real-time PCR analysis. XZ drew the comparative synteny map. XY grew the plants and performed experiments including collecting RNA and synthesizing cDNA. KLR, CD, RWS carried out the biomass composition analysis and MBMS analysis under the supervision of MD. NRS assisted in the real-time PCR analysis. RDS, PG, JB, and WS helped to grow *Arabidopsis *mutants and performed molecular analysis. XY provided important suggestions for phylogenetic analysis. JB and NL provided important suggestions on biomass analysis. DH provided suggestions on biomass analysis and carried out preliminary analysis. ZMC supervised XY's work. CNS Jr. had oversight over a portion of the work and helped to draft the manuscript.

## Supplementary Material

Additional file 1 Phylogenetic analysis C4H genes across different species. The naming of the genes follows the schema in Figure 2 to 4. The analysis indicated that C4H genes from lower plants share a clade, while there is no distinction between monocot and dicot species. Click here for file

Additional file 2 Phylogenetic analysis of COMT gene family. The analysis reviews three major groups as lower plants, monocot and dicot. Most of the COMT genes followed the group classification in the phylogenetic analysis. Click here for file

Additional file 3 Phylogenetic analysis of CCR gene family. The analysis revealed three major clades for each group of plants as monocot, dicot, and lower plants. Click here for file
