# Supplementary figures and images for: Comparative genome analysis of lignin biosynthesis gene families across the plant kingdom
Source: BMC Bioinformatics. 2009 Oct 8;10(Suppl 11):S3. doi: 10.1186/1471-2105-10-S11-S3 (PMC3226193; doi:10.1186/1471-2105-10-S11-S3)

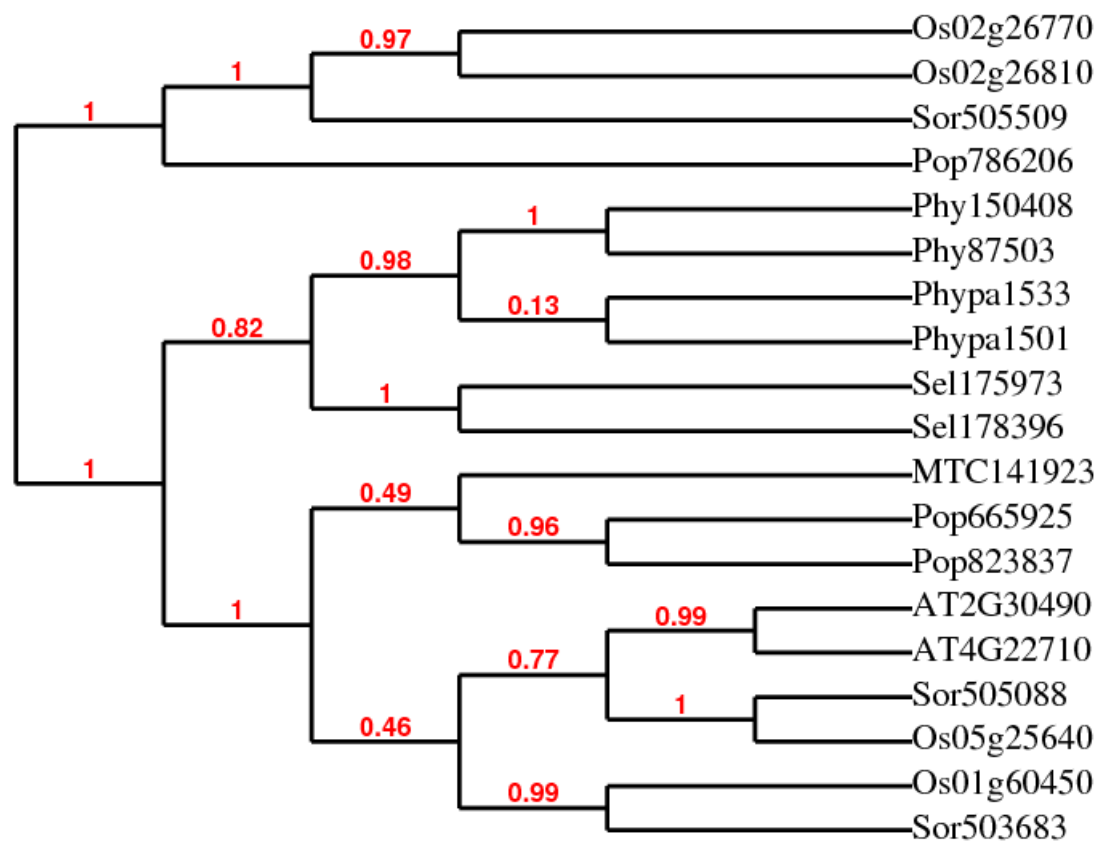

Supplement: Additional file 1 — Phylogenetic analysis C4H genes across different species. The naming of the genes follows the schema in Figure 2 to 4. The analysis indicated that C4H genes from lower plants share a clade, while there is no distinction between monocot and dicot species. [file 1471-2105-10-S11-S3-S1.pdf]

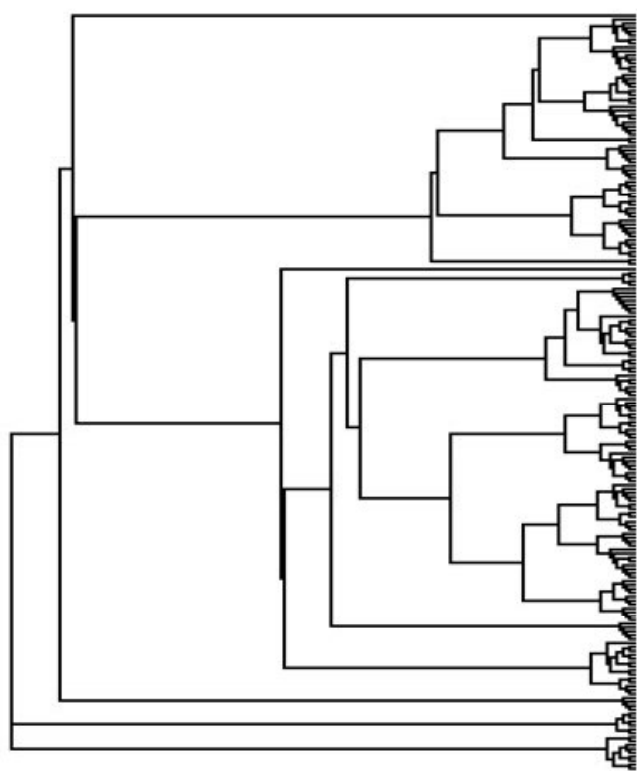

**Dicots**

**Monocots**

**Spike Moss and  
Physcomettrila**

Supplement: Additional file 2 — Phylogenetic analysis of COMT gene family. The analysis reviews three major groups as lower plants, monocot and dicot. Most of the COMT genes followed the group classification in the phylogenetic analysis. [file 1471-2105-10-S11-S3-S2.pdf]

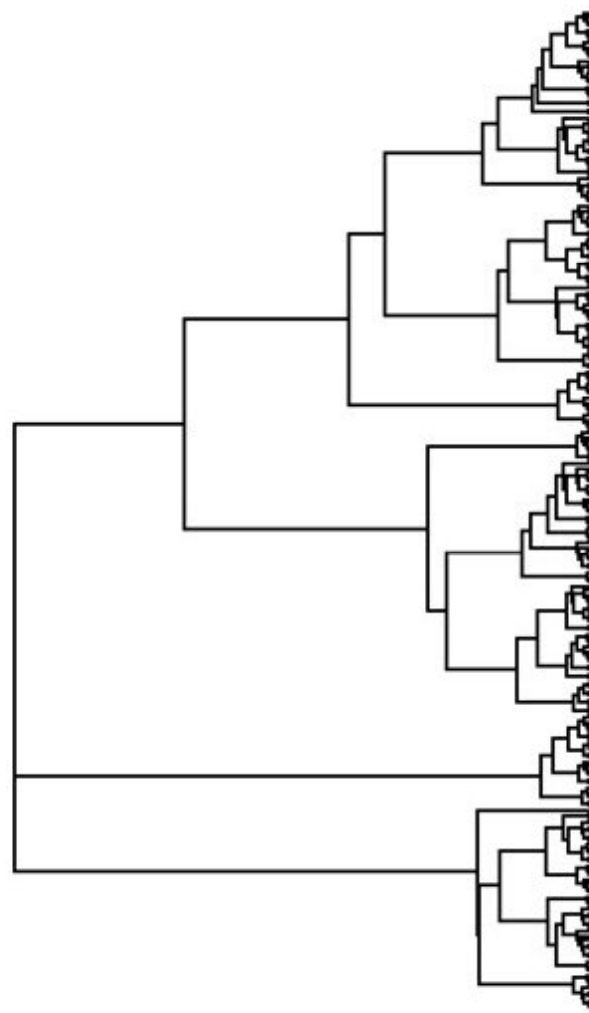

**Monocots**

**Dicots**

**Lower species**

Supplement: Additional file 3 — Phylogenetic analysis of CCR gene family. The analysis revealed three major clades for each group of plants as monocot, dicot, and lower plants. [file 1471-2105-10-S11-S3-S3.pdf]
